# Decreased expression of the clock gene Bmal1 is involved in the pathogenesis of temporal lobe epilepsy

**DOI:** 10.1186/s13041-021-00824-4

**Published:** 2021-07-14

**Authors:** Hao Wu, Yong Liu, Lishuo Liu, Qiang Meng, Changwang Du, Kuo Li, Shan Dong, Yong Zhang, Huanfa Li, Hua Zhang

**Affiliations:** 1 Department of Neurosurgery, Clinical Research Center for Refractory Epilepsy of Shannxi Province, The First Affiliated Hospital of Xi’an Jiaotong University, 277 West Yanta Road, Xi’an, 710061 Shaanxi China; 2 Center for Mitochondrial Biology and Medicine, The Key Laboratory of Biomedical Information Engineering of Ministry of Education, School of Life Science and Technology, School of Life Science and Technology, Xi’an Jiaotong University, Xi’an, Shaanxi, China; 3 Center of Brain Science, The First Affiliated Hospital of Xi’an Jiaotong University, 277 West Yanta Road, Xi’an, 710061 Shaanxi China; 4 School of Basic Medical Sciences, Xi’an Jiaotong University Health Science Center, Xi’an, Shaanxi China

**Keywords:** Clock genes, Bmal1, Protocadherin 19, Dentate gyrus, Temporal lobe epilepsy, Transgenic mice

## Abstract

Clock genes not only regulate the circadian rhythm of physiological activities but also participate in the pathogenesis of many diseases. Previous studies have documented the abnormal expression of clock genes in epilepsy. However, the molecular mechanism of brain and muscle Arnt-like protein 1 (Bmal1), one of the core clock genes, in the epileptogenesis and seizures of temporal lobe epilepsy (TLE) remain unclear. We first investigated the levels of Bmal1 and other clock proteins in the hippocampus of subjects with epilepsy to define the function of Bmal1. The levels of Bmal1 were decreased during the latent and chronic phases in the experimental group compared with those in the control group. Knockout of Bmal1 in hippocampal dentate gyrus (DG) neurons of Bmal1^flox/flox^ mice by Synapsin 1 (Syn1) promoter AAV (adeno-associated virus) lowered the threshold of seizures induced by pilocarpine administration. High-throughput sequencing analysis showed that PCDH19 (protocadherin 19), a gene associated with epilepsy, was regulated by Bmal1. PCDH19 expression was also decreased in the hippocampus of epileptic mice. Furthermore, the higher levels of Bmal1 and PCDH19 were detected in patients with no hippocampal sclerosis (no HS) than in patients with HS International League Against Epilepsy (ILAE) type I and III. Altogether, these data suggest that decreased expression of clock gene Bmal1 may participate in epileptogenesis and seizures via PCDH19 in TLE.

**Supplementary Information:**

The online version contains supplementary material available at 10.1186/s13041-021-00824-4.

## Introduction

Temporal lobe epilepsy (TLE) is a common form of drug-resistant epilepsy. The common pathological change in TLE is hippocampal sclerosis (HS), which is characterized by severe neuronal loss and gliosis in one or more hippocampal regions [1]. Seizures in patients with TLE are often accompanied by cognitive impairment, memory loss, and mood impairments. Clinical observations have shown that patients with TLE exhibit a 24-h nonuniform distribution of seizure occurrence, which may present unimodal (afternoon) or bimodal (morning and noon) temporal peaks [2–4]. Spontaneous seizures in kainic acid/pilocarpine-induced and electrically stimulated models of TLE occur in a pattern of a 24-h nonuniform distribution [5, 6]. Based on these data, seizures in TLE may be associated with circadian rhythms.

Circadian rhythms are biological rhythms driven by a series of clock genes, such as Bmal1 (brain and muscle Arnt-like protein 1) and CLOCK (circadian locomotor output cycles kaput) [7]. As the core clock genes, Bmal1 and CLOCK in the cytoplasm heterodimerize and translocate to the nucleus and then instigate transcription of target genes by interacting with E-box promoters. Among these target genes, some clock genes, such as Period (Per1/2/3) and Cryptochrome (Cry1/2), are involved in the positive and negative regulation of the circadian oscillation of Bmal1 and CLOCK. The downstream genes of the clock gene are called as Clock-controlled genes (CCGs), such as D-element binding protein (Dbp) and E4 binding protein 4 (E4bp4) [8, 9]. Clock genes and CCGs underlie the rhythmic oscillations at a cellular and organismal level [10]. In the epileptic hippocampus, the levels of Bmal1, CLOCK, Cry and Per mRNAs have been confirmed to be decreased. Previous studies have shown that the expression levels of clock genes change after seizures and can regulate downstream genes (such as Slc6a1 and Slc6a11) to directly affect epileptogenesis [9, 11, 12]. Deletion of a single or multiple clock genes and CCGs is associated with an increased susceptibility to seizures in mice. However, the molecular mechanisms underlying circadian clock-controlled temporal patterns of epileptic activity and seizures remain unclear.

Several studies have investigated the role of Bmal1 in the astrocyte activation, circadian clock function and neurodegenerative disease, such as Alzheimer’s disease [13–15]. Bmal1 mRNA expression is decreased at *Zeitgeber time* 12 (ZT12) in hippocampus of pilocarpine-treated rats (a model of TLE) [16]. The threshold of seizures induced by electrical stimulation in Bmal1 knockout (KO) mice is lower than in controls [17]. Notably, in the study by Ferraro, the ablation of Bmal1 was not limited to specific brain nuclei and cellular types. Subsequent studies find that the ablation of Bmal1 in GLAST (glutamate/aspartate transporter)-positive astrocytes in the suprachiasmatic nucleus (SCN) alters circadian locomotor behavior and cognition in mice through GABA signaling [18]. Moreover, the deletion of Bmal1 in astrocytes induces astrocyte activation and inflammatory gene expression via a cell-autonomous mechanism [14]. Based on these findings, abnormal expression of Bmal1 may be related to the pathogenesis of TLE. However, researchers have not clearly determined how changes in Bmal1 expression in the hippocampus, especially in hippocampal neurons, affect the epileptogenesis and seizures in individuals with TLE.

Therefore, in this study, we explored the potential role of Bmal1 in epileptic activity and seizures. We found that the expression of Bmal1 was decreased in the hippocampus of subjects with TLE. Neuron-specific knockout of Bmal1 in Dentate gyrus (DG) neurons of Bmal1^flox/flox^ mice increased the susceptibility and mortality rate from seizures induced by pilocarpine treatment. Downstream genes regulated by Bmal1 were identified through high-throughput sequencing. The expression level of PCDH19 was verified and shown to be gradually decreased in the hippocampus after the seizures. Moreover, expression levels of Bmal1 and PCDH19 were more decreased to a greater extent in the hippocampal with HS groups than in that of the no HS group.

## Materials and methods

### Patient selection

The data and specimens from patients included in the study were obtained from the files of the Department of Neurosurgery, The First Affiliated Hospital of Xi'an Jiaotong University. We examined 16 specimens obtained from patients undergoing surgery for medically intractable TLE. All procedures were performed with the informed consent of the patients or legal next-of-kin and were approved by the Committee on Human Research at The First Affiliated Hospital of Xi'an Jiaotong University. Epilepsy was diagnosed according to the 2017 International Classification of Epileptic Seizures by the International League Against Epilepsy. Antiseizure drug therapy failed in all patients treated with maximum doses of at least three antiseizure drugs, including valproic acid, carbamazepine, phenytoin sodium, phenobarbital, clonazepam, topiramate, gabapentin, lamotrigine, and oxcarbazepine. Before surgery, patients were evaluated by using multiple methods, including high-resolution magnetic resonance imaging (MRI), positron emission tomography (PET), long-term video electroencephalogram (EEG), and/or intraoperative electrocorticography (ECoG). Some of the patients underwent chronic intracranial EEG monitoring. Two neuropathologists reviewed all of the specimens independently. Additional file 1: Table 1 summarizes the patients’ clinical features.

### Animals

Adult C57/BL6J mice weighing 20–25 g were purchased from the experimental animal center of Medical College at Xi’an Jiaotong University. Bmal1^flox/flox^ mice were purchased from The Jackson Laboratory (Stock No: 007668). The animals were housed under controlled humidity (55 ± 5%) and temperature (20 ± 2 °C) conditions with a normal 12-h light/12-h dark cycle. Water and food were available ad libitum. All animal procedures were carried out in line with the National Institutes of Health Guide for the Care and Use of Laboratory Animals and were approved by the Institutional Animal Care and Use Committee. All procedures performed in studies involving animals were approved by the Ethics Committee of Xi’an Jiaotong University (Ethics and Science # G-83) in full accordance with the ethical guidelines of the National Institutes of Health for the care and use of laboratory animals. All efforts were made to minimize suffering and the number of mcie used in the experiments.

### Study design and experimental endpoints

To investigate the levels of proteins after seizures, three of 20 mice were randomly taken out and administered saline vehicle as a control group, and the remaining mice were administered pilocarpine to induce seizures. Based on the Racine categories, 14 mice had seizures that reached Racine category 4 and were included in the epilepsy model group. Subsequently, 3 mice were randomly euthanized at different time points (1 day, 3 days, 14 days, 60 days). Each mouse brain was divided into two halves along the longitudinal fissure. One half was used for immunoblotting experiments. The other half was fixed with 4% paraformaldehyde solution in PBS and used for immunofluorescence staining. To evalute the effects of Bmal1 conditional KO (cKO) on susceptibility to seizures, twenty-six Bmal1^flox/flox^ mice were randomly divided into 2 groups and injected Syn1-mCherry and Syn1-Cre AAVs respectively. Four weeks after the AAVs injection, 3 mice were randomly selected from each group of animals to test the efficiency of Bmal1 cKO and transcriptome sequencing. Behavioral tests of 20 mice from the two groups were evaluated based on Racine categories. After behavioral testing, mice were sacrificed, and samples were collected samples for immunoblotting and immunofluorescence experiments.

### Pilocarpine treatment and seizure assessment

Mice were intraperitoneally administered methylscopolamine (1 mg/kg body weight) 30 min before the injection of pilocarpine (300 mg/kg body weight) in 0.2 ml of sterile saline vehicle (0.9% NaCl). Control mice received an equivalent volume of saline vehicle. The severity of seizure behavior was observed for 1 h and assessed using the standard described below. Categories 1–2 involved one or more of the symptoms, which included facial automatisms, tail stiffening, and wet-dog shakes. Categories 1 and 2 were considered as a group to avoid subjectivity in assessing the seizures. Category 3 involved clonic unilateral forelimb myoclonus in addition to the symptoms listed above. Category 4 involved bilateral forelimb myoclonus and rearing. Category 5 involved generalized clonic-tonic convulsions and loss of postural control. The seizure categories were separately evaluated by two observers. A mouse experiencing continuous category 3–5 seizure events (30–90 s) was considered to have undergone status epilepticus (SE). Diazepam (10 mg/kg, i.p.) was administered to terminate SE 1 h after the onset. Mice in categories 3–5 were monitored for 2 h/day, 7 days/week for the occurrence of spontaneous seizures. Only after the occurrence of a seizure was a mouse identified as having epilepsy (i.e. the observer was not aware of priori treatment administered to any mouse). Methylscopolamine (S1978) and pilocarpine (S4231) were purchased from Selleck. Diazepam was purchased from Shanghai Shyndec Pharmaceutical Co., Ltd.

### Total protein preparation and immunoblotting

All mice were sacrificed under deep anesthesia with isoflurane (VETEASY, RWD Life Science). Then, the skull was opened and the brain tissue was carefully removed. The brain tissue was placed in precooled PBS, and the hippocampus was separated from the remaining brain tissue with fine tweezers. Hippocampal tissues were homogenized in radioimmunoprecipitation assay (RIPA) buffer (25 mM Tris·HCl at pH 7.6, 150 mM NaCl, 0.1% SDS, 1% NP-40, 1% sodium deoxycholate, and protease inhibitor (ThermoFisher, 89901)). The homogenates were then centrifuged at 12,000 rpm for 20 min at 4 °C. The protein concentration of the tissue lysate was measured with the bicinchoninic acid (BCA) assay (Thermo Fisher Scientific, 23227) according to the manufacturer’s protocol. Protein lysates were mixed with a one-third volume of 4 × loading buffer. The supernatants were boiled and separated by sodium dodecyl sulfate–polyacrylamide gel electrophoresis (SDS-PAGE). Proteins were electrophoresed and transferred to PVDF filter membranes (Merck Millipore, ISEQ00010). The membranes were blocked with TBST (0.1% Tween 20) containing 5% nonfat milk powder (w/v) for 1 h then incubated with primary antibodies. Corresponding HRP-conjugated secondary antibodies were subsequently incubated with the membrane for 2 h at room temperature. Protein bands were detected with chemiluminescence using a GenoSens 2000 imaging system (Clinx Science Instruments Co., Ltd.). The following primary and secondary antibodies were used: anti-Bmal1 (Abcam, ab93806), anti-β-actin (Proteintech, 66009-1-Ig), anti-CLOCK (Abcam, ab3517), anti-Per2 (Abcam, ab179813), anti-Cry1 (Proteintech, 13474-1-AP), anti-NeuN (Merck Millipore, ABN90), anti-PCDH19 (Abclonal, A10067), anti-PCDH19 (Abcam, ab191198), anti-Cre (Cell Signaling Technology, 15036), HRP-conjugated Affinipure Goat Anti-Mouse IgG(H + L) (Proteintech, SA00001-1), HRP-conjugated Affinipure Goat Anti-Rabbit IgG(H + L) (Proteintech, SA00001-2).

### Immunofluorescence staining

Mice were anesthetized with isoflurane (VETEASY, RWD Life Science) and transcardially perfused with phosphate-buffered saline (PBS) followed by freshly prepared 4% polyformaldehyde. Brains were then removed, postfixed for 6 h, and gradient dehydrated in 20 and 30% sucrose in PBS. For each brain, 12-μm-thick coronal sections were cut and stored at − 20 °C. Samples were permeabilized with 1% Triton X-100 in PBS for 15 min. Incubate samples with 10% normal goat serum in PBS for 30 min at room temperature. Sections were incubated with the following primary antibodies at 4 °C overnight: anti-Bmal1 (Abcam, ab93806), anti-NeuN (Merck Millipore, ABN90), and anti-PCDH19 (Abclonal, A10067). The sections were rinsed three times with PBS for 5 min each and then stained with the corresponding fluorescent dye-conjugated secondary antibodies for 3 h at room temperature. Goat Anti-Guinea pig IgG H&L (Alexa Fluor® 488) (Abcam, ab150185); Goat Anti-Rabbit IgG H&L (Cy3) (Abcam, ab6939) were used. Finally, sections were mounted and imaged using an OLYMPUS BX53 microscope.

### Stereotaxic injection of adeno-associated virus (AAV)

Mice were anesthetized with isoflurane (VETEASY, RWD Life Science), with a concentration of 4% during anesthesia induction and 1.5–2.5% as the maintenance level, and then positioned in the stereotaxic instrument. A craniotomy was performed using a hand-held drill. A glass micropipette was used to deliver 300 nL AAV (1E + 13 VG/mL) into bilateral hippocampal dentate gyrus (DG) areas (bregma coordinates: anteroposterior − 2.40 mm; mediolateral ± 2.00 mm; dorsoventral − 2.20 mm) within 60 s. rAAV2/9-hSyn-Cre-mCherry-WPRE-pA and rAAV2/9-hSyn-mCherry-WPRE-pA were purchased from BrainVTA (BrainVTA Co., Ltd., China).

### High throughput sequencing (RNA sequencing analysis)

Mice were anesthetized with isoflurane (VETEASY, RWD Life Science) and transcardially perfused with phosphate-buffered saline (PBS). The brain tissue was placed in pre-cooled PBS, and the hippocampus was separated from the remaining brain tissue with fine tweezers. Total RNA of the hippocampus was extracted by an RNeasy Mini Kit (QIAGEN, 74106). RNA samples were then further purified with magnetic oligo(dT) beads after denaturation. Purified mRNA samples were reverse transcribed into first-strand cDNAs, and a second cDNAs were further synthesized. Fragmented DNA samples were blunt-ended and adenylated at the 3′ ends. Adaptors were ligated to construct a library. DNA consertration was quantified using Qubit (Invitrogen). After cBot cluster generation, DNA samples were then sequenced on an Illumina Hiseq2500 SBS platform from Genergy Biotechnology Co., Ltd. (Shanghai, China). Raw data were converted into Fastq format. The number of transcripts in each sample was calculated based on the number of fragments per kilobase of transcript per million fragments mapped (FPKM); Cuffnorm software was used to calculate the FPKM value for each sample, and the values were log2 transformed. DESeq2 software was used to calculate the differential gene expression between different samples. FDR (adjusted p-value) ≤ 0.05 was used to identify upregulated or downregulated RNAs. For the KEGG pathway analysis, the entire set of genes was used as the background list, the differentially expressed genes were used as the candidate list, and *p* values *were* calculated. Significant genes were categorized based on gene functions. Data analysis was performed at Genergy Biotechnology Co., Ltd. (Shanghai, China).

### Statistical analysis

Differences in protein expression levels at different time points and the fluorescence intensity among three groups were analyzed using one-way ANOVA. Differences in protein expression levels and fluorescence intensities between the two groups were analyzed using Student’s *t*-test. Data on the mortality rate were compared using Fisher’s exact test. The data are presented as the mean ± standard error of the mean (SEM). Statistical significance was set at p < 0.05. The detailed statistical tests used for each analysis are stated in the figure legends. All statistical analyses were performed with RStudio software (version 1.3.1093; https://rstudio.com/products/rstudio/).

## Results

### Dynamic changes in clock genes in the hippocampus after seizures

**Fig. 1:**
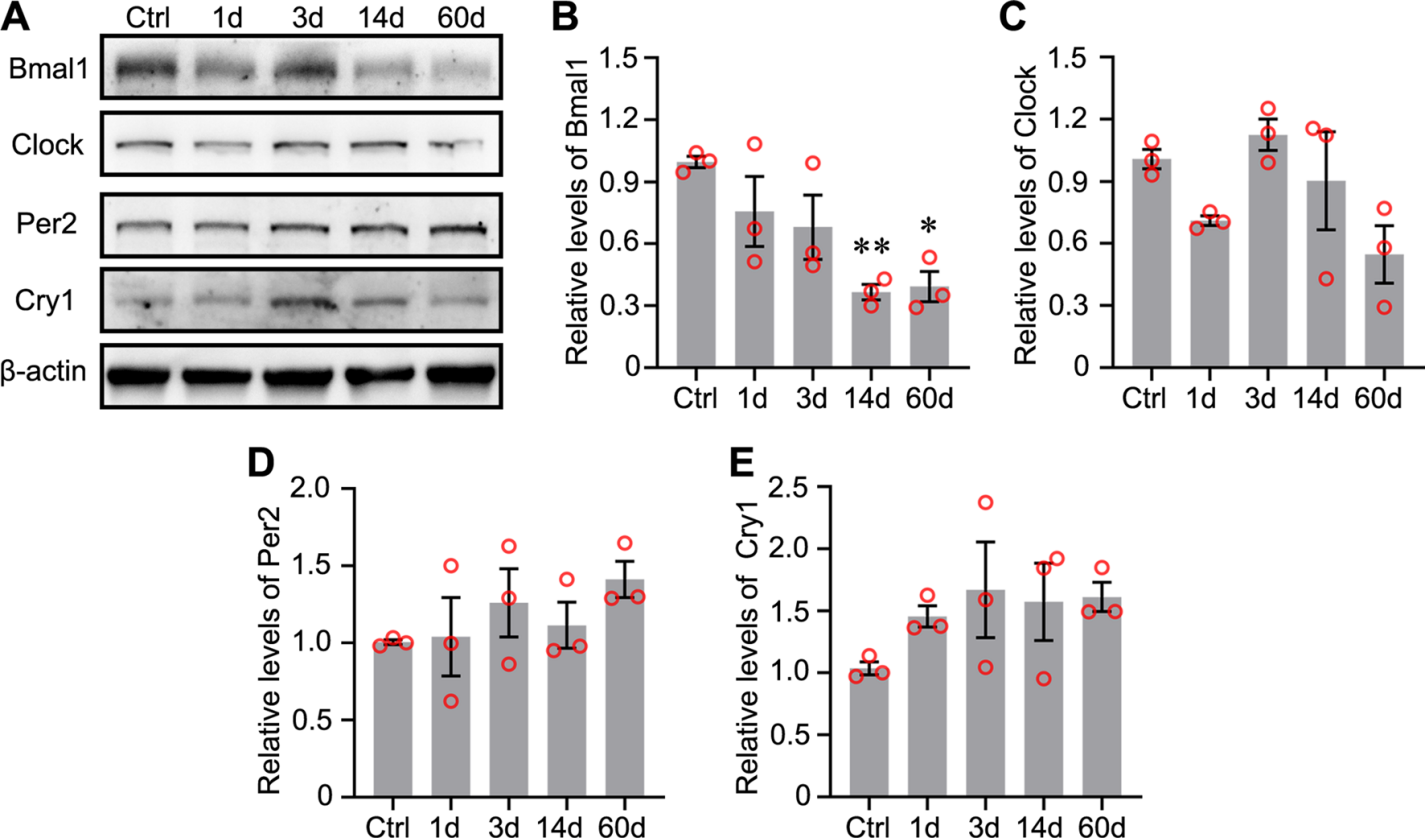
Expression of Circadian rhythm proteins in mouse hippocampus following pilocarpine-induced status epilepticus (SE). **A** Changes in protein levels of Circadian rhythm proteins at different time points following pilocarpine-induced SE. **B**–**E** Comparison of Bmal1 blots density between control mice and epileptic mice at each time point after SE (n = 3 per group). Bmal1 expression was significantly decreased at 14 days (0.366 ± 0.038) and 60 days (0.393 ± 0.073), compared with Ctrl (0.995 ± 0.027). There is no significant difference in the Clock, Per2, and Cry1 expression levels between the control group and epileptic groups at different time points. The data are expressed as mean ± SEM and analyzed with one-way ANOVA. *p < 0.05, **p < 0.01

**Fig. 2:**
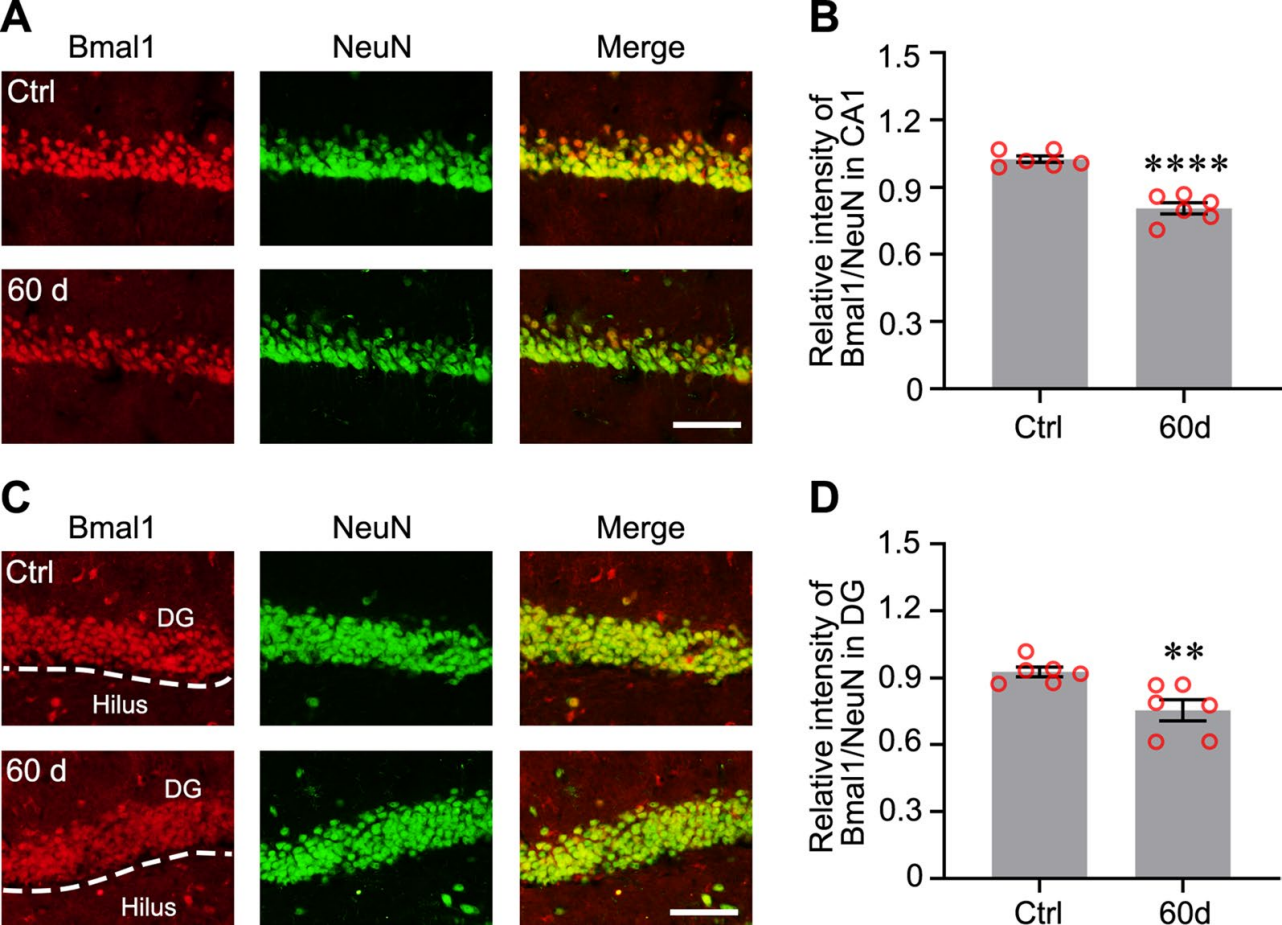
Immunofluorescence detection of Bmal1 expression in the hippocampal CA1 and DG of epileptic mice. **A** Detection of the endogenous Bmal1 protein (red) in CA1 by immunofluorescent labeling. Neurons were labeled by the neuronal marker, NeuN (green). Scale bar, 100 μm. **B** Analysis of fluorescence intensity was performed using ImageJ. Differences in the relative fluorescence intensity (Bmal1 vs. NeuN) were analyzed with the Students *t*-test (Control: 1.025 ± 0.015, 60 days: 0.805 ± 0.025, n = 3, *p* < 0.0001). **C** Detection of Bmal1 protein (red) and NeuN (green) in DG by immunofluorescent labeling. Scale bar, 100 μm. **D** Analysis of fluorescence intensity was performed using ImageJ. Differences in the relative fluorescence intensity (Bmal1 vs. NeuN) were analyzed with the Students *t*-test (Control: 0.926 ± 0.022, 60 days: 0.755 ± 0.047, n = 3, *p* = 0.008). The data are expressed as mean ± SEM and analyzed with unpaired Student’s *t*-test. **p < 0.01, ***p < 0.001

Previous studies have reported the changes in mRNA expression levels of some clock genes [16], but changes in the expression of the proteins encoded by these genes in the hippocampus have not been clearly elucidated. We first detected the expression levels of clock proteins at different time points to study the role of clock proteins in the TLE. The acute phase was defined as 1–3 days after SE [19]. The latent phase was defined as a seizure-free period that can last for weeks [20, 21]. The chronic phase was defined as the period during which mice exhibited spontaneous, recurrent seizures. Bmal1 expression was decreased in the latent phase (14 days post-SE) and chronic phase (60 days post-SE) in the experimental group compared with the control group (Fig. 1A and B). Clock expression was decreased in the acute (1 day post-SE) and chronic phase, although one-way ANOVA analysis did not reveal statistically significant differences (Fig. 1C). Statistically significant changes in the levels of Per2 and Cry1 protein were not observed (Fig. 1D, E). Because the bulk sample uesd for immunoblotting detection cannot clarify which subregions of the hippocampus showed decreased Bmal1 expression. The abnormal distribution of Bmal1 in the hippocampus was detected using immunofluorescence staining. Compared to control, the seizure group showed a reduced fluorescence intensity of Bmal1 staining was in the CA1 and dentate gyrus (DG) during the chronic phase (Fig. 2A–D).

### Conditional knockout of Bmal1 in DG neurons increased the susceptibility to seizures

**Fig. 3:**
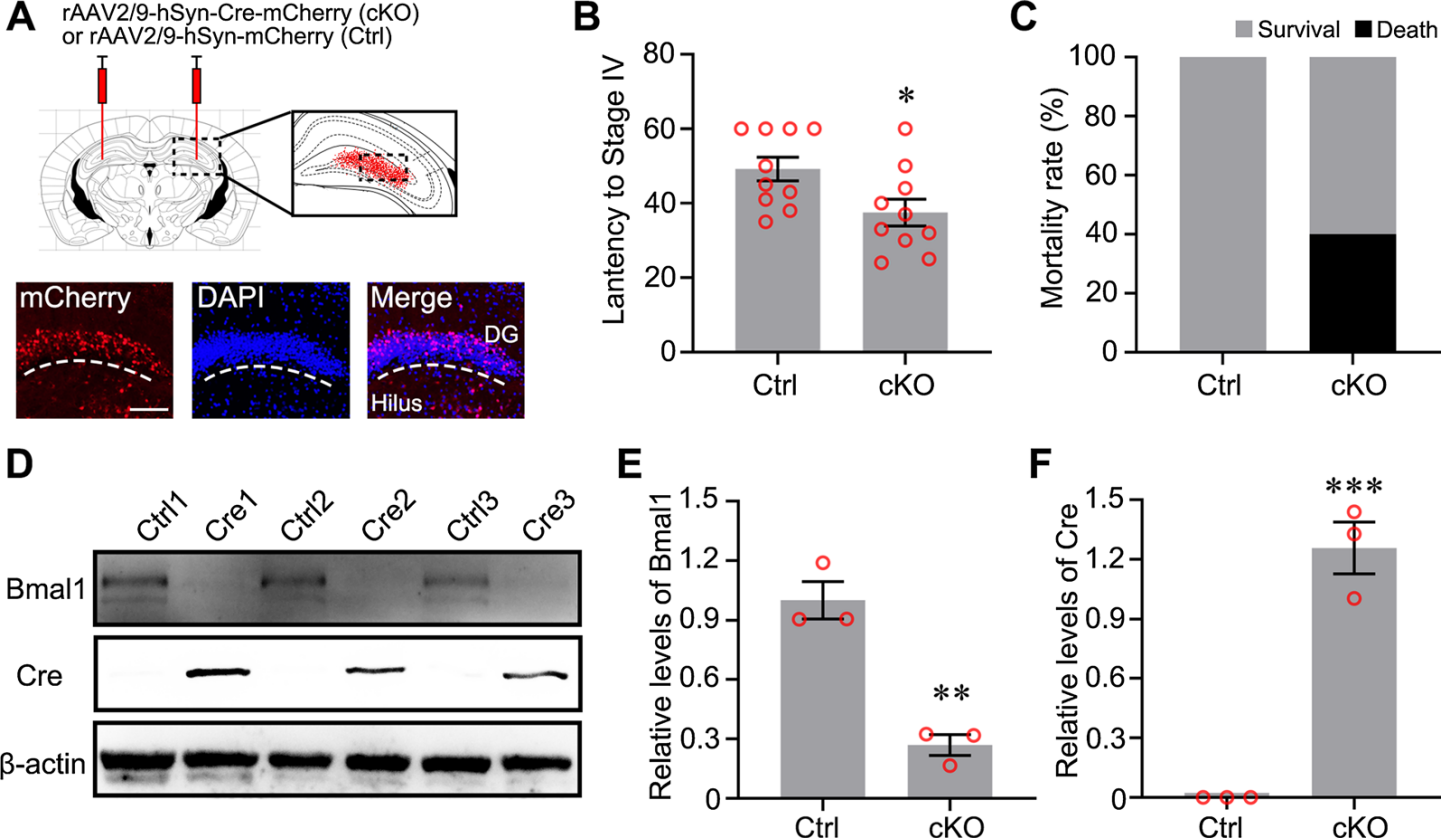
Conditional knockout (cKO) of Bmal1 in DG neurons increased the susceptibility to seizures. **A** Schematic diagram of AAV injection and the expression of mCherry encoded by an AAV carrying mCherry in DG neurons. Scale bar, 100 μm. **B** Seizure behavior assessment in control group (n = 10) and cKO group (n = 10) after pilocarpine administration. Data of latency (control: 49.20 ± 3.193; cKO: 37.50 ± 3.583, *p* = 0.025) are presented as mean ± SEM and analyzed using unpaired Student’s *t*-test. **C** Data of mortality rate in the control group and cKO group (n = 10, *p* = 0.094) were carried out using Fisher’s exact test. **D** The efficiency of Bmal1 cKO in Bmal1^flox/flox^ mice verified by immunoblotting. Bmal1 expression was significantly decreased at the cKO group, compared with the Ctrl group. Differences in the levels of Bmal1 are presented as mean ± SEM and analyzed using unpaired Student’s *t*-test (control: 1.000 ± 0.095, 0.270 ± 0.052, n = 3, *p* = 0.003). **E** Cre expression was significantly increased at the cKO group, compared with the Ctrl group. Differences in the levels of Cre are presented as mean ± SEM and analyzed using unpaired Student’s *t*-test (control: 0, cKO: 1.257 ± 0.131, n = 3, *p* = 0.0007). *p < 0.05, **p < 0.01, ***p < 0.001

To clarify the effect of decreased Bmal1 on epileptogenesis, AAV2/9-hSyn-Cre-mCherry or control virus were injected into the bilateral hippocampal DG area of Bmal1^flox/flox^ mice (Fig. 3A). Neuron-specific knockout of Bmal1 (Bmal1 cKO) in DG significantly shortened the latency for seizures (Fig. 3B). Latency refers to the time from the pilocarpine administration (i.p.) to seizures of Racine category 4 [22]. Although the difference was not statistically significant, Bmal1 cKO increased the mortality rate resulted from seizures (Fig. 3C). The efficiency of Bmal1 cKO in Bmal1^flox/flox^ mice was verified by immunoblotting. Compared with the control virus group, Bmal1 protein expression was significantly decreased in the cKO group (Fig. 3D, E), while Cre protein expression was significantly up-regulated in the cKO group (Fig. 3D–F).

### Protocadherin 19 (PCDH19) as a potential candidate gene regulated by Bmal1

**Fig. 4:**
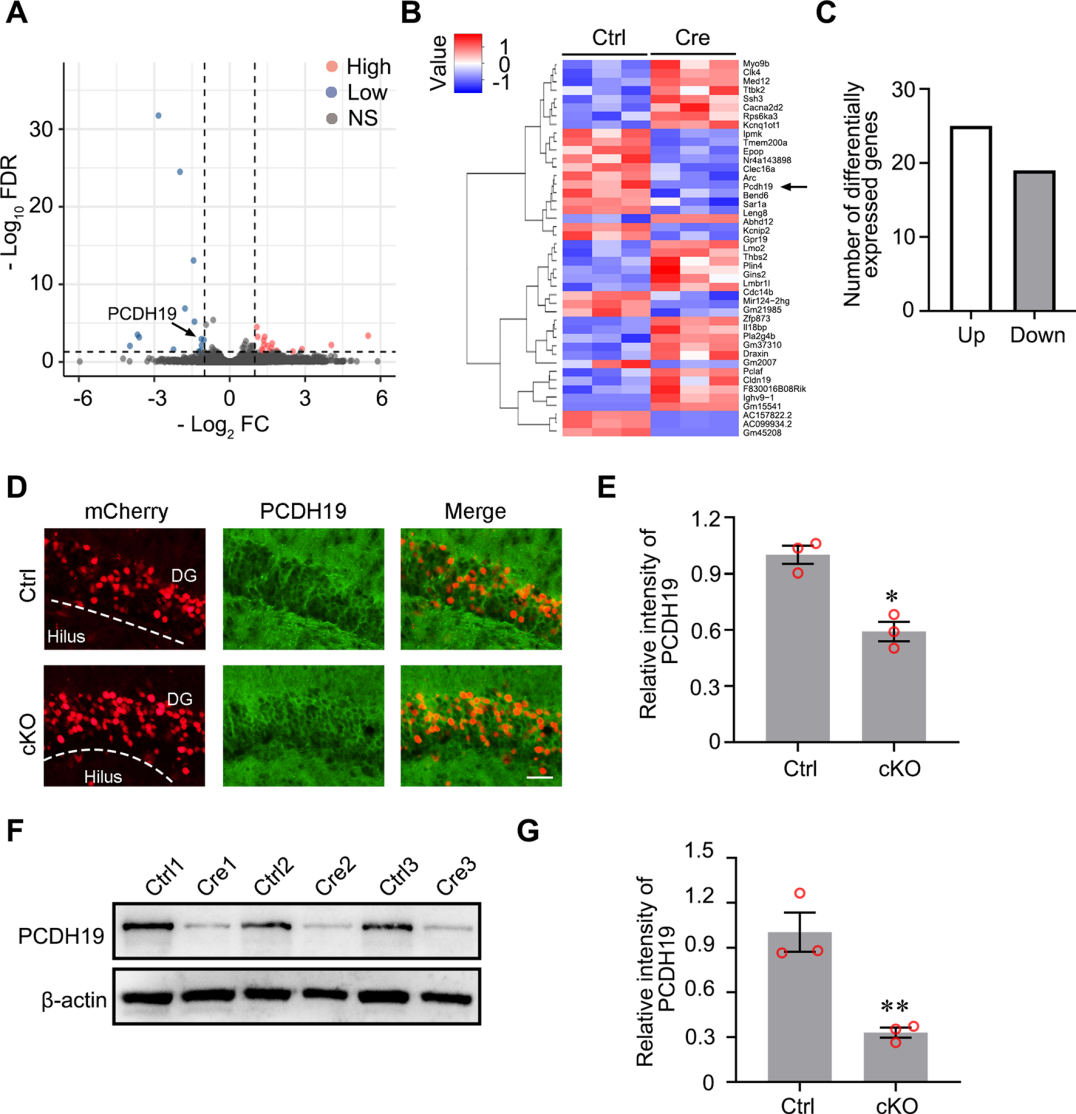
PCDH19 as a potential candidate gene regulated by Bmal1. **A** The volcano plot of differentially expressed genes between the control group and the cKO group (FDR < 0.05). **B** Heat map of significantly up- or down-regulated genes between the control group and the cKO group. Each group contained three batches of individual samples. **C** The number of the significantly differentially expressed genes between the control group and the cKO group (fold change > 2, FDR < 0.05). **D**, **E** Immunofluorescence detection of mCherry and PCDH19 expression in DG of mice. Scale bar, 50 μm. Analysis of fluorescence intensity was performed using ImageJ. Differences in the intensity between the control group and the cKO group were analyzed with the Students *t*-test (Control: 1.000 ± 0.049, cKO: 0.592 ± 0.052, n = 3, *p* = 0.007). **F**, **G** PCDH19 expression was significantly decreased at the cKO group, compared with the Ctrl group. Differences in the levels of PCDH19 are presented as mean ± SEM and analyzed using unpaired Student’s *t*-test (control: 1.003 ± 0.131, cKO: 0.330 ± 0.033, n = 3, *p* = 0.008). The data are expressed as mean ± SEM and analyzed with unpaired Student’s *t*-test. *p < 0.05, **p < 0.01

To further clarify the mechanism by which Bmal1 cKO increased the susceptibility to seizures induced by pilocarpine administration, the hippocampal tissues from Bmal1 cKO and control tissues were subjected to high-throughput sequencing. An FDR (adjusted p-value) ≤ 0.05 was used to identify upregulated or downregulated mRNAs between the different samples. The top 25 up-regulated and 19 down-regulated genes are displayed in a volcano plot and heatmap plot (Fig. 4A–C). Among these genes, PCDH19 has been reported to be closely related to epilepsy [23]. Mutations in this gene on human chromosome X are associated with sporadic infantile epileptic encephalopathy and a female-restricted form of epilepsy [24]. PCDH19 was detected in the brain tissue slices from Bmal1 cKO mice (Fig. 4D). The fluorescence intensity of PCDH19 was significantly decreased in the cKO group compared with the control group (Fig. 4E). The expression level of PCDH19 was also downregulated in the cKO group compared with the control group (Fig. 4F, G).

### Decreased expression of PCDH19 in the hippocampus of epileptic mice

**Fig. 5:**
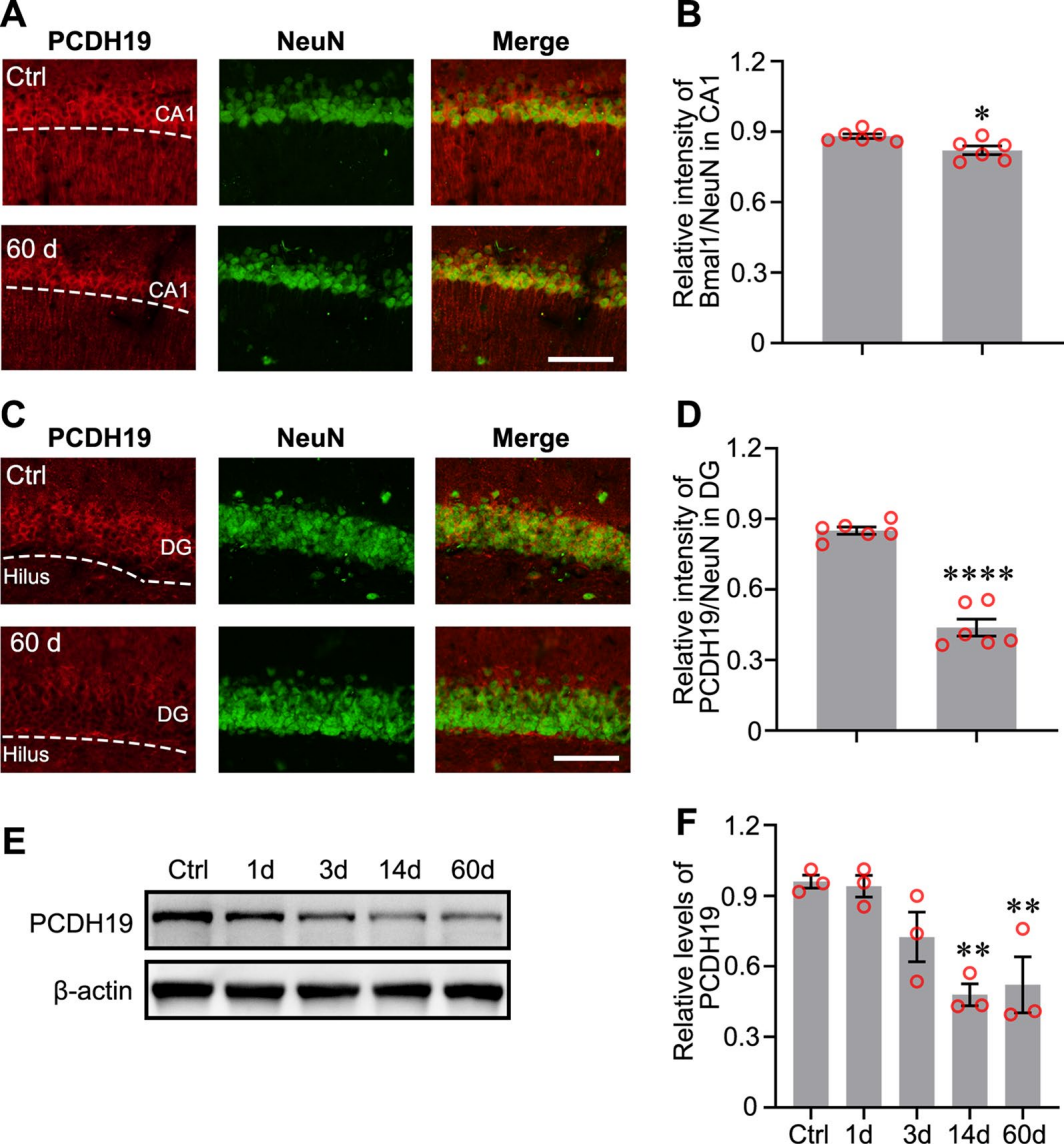
PCDH19 expression in the hippocampal CA1 and DG of epileptic mice. **A** Detection of the endogenous PCDH19 protein (red) in CA1 by immunofluorescent labeling. Neurons were labeled by the neuronal marker, NeuN (green). Scale bar, 100 μm. **B** Analysis of fluorescence intensity was performed using ImageJ. Differences in the relative fluorescence intensity (PCDH19 vs. NeuN) were analyzed with the Students *t*-test (Control: 0.987 ± 0.006, 60 days: 0.947 ± 0.012, n = 3, *p* = 0.015). **C** Detection of PCDH19 protein (red) and NeuN (green) in DG by immunofluorescent labeling. Scale bar, 100 μm. **D** Analysis of fluorescence intensity was performed using ImageJ. Differences in the relative fluorescence intensity (PCDH19 vs. NeuN) were analyzed with the Students *t*-test (Control: 0.967 ± 0.010, 60 days: 0.693 ± 0.024, n = 3, *p* < 0.0001). The data are expressed as mean ± SEM and analyzed with unpaired Student’s *t*-test. **p < 0.01, *p < 0.05, ****p < 0.0001. **E** The levels of PCDH19 protein at different time points following pilocarpine-induced SE. **F** Comparison of PCDH19 blots density between control mice and epileptic mice at each time point after SE (n = 3 per group). Bmal1 expression was significantly decreased at 14 days (0.480 ± 0.046) and 60 days (0.522 ± 0.119), compared with Ctrl (0.960 ± 0.028). The data are expressed as mean ± SEM and analyzed with one-way ANOVA, **p < 0.01

Although PCDH19 mutations cause epilepsy, the expression of PCDH19 in individuals with acquired epilepsy has not been reported. Firstly, the distribution of PCDH19 in the hippocampus of TLE mice was detected using immunofluorescence staining. Compared to controls, the fluorescence intensity of PCDH19 staining was faint in the CA1 and DG during the chronic phase (Fig. 5A–D). The levels of the PCDH19 protein were also decreased in the latent phase and the chronic phase (Fig. 5E, F).

### Abnormal expression of Bmal1 and PCDH19 in patients with TLE with hippocampal sclerosis

**Fig. 6:**
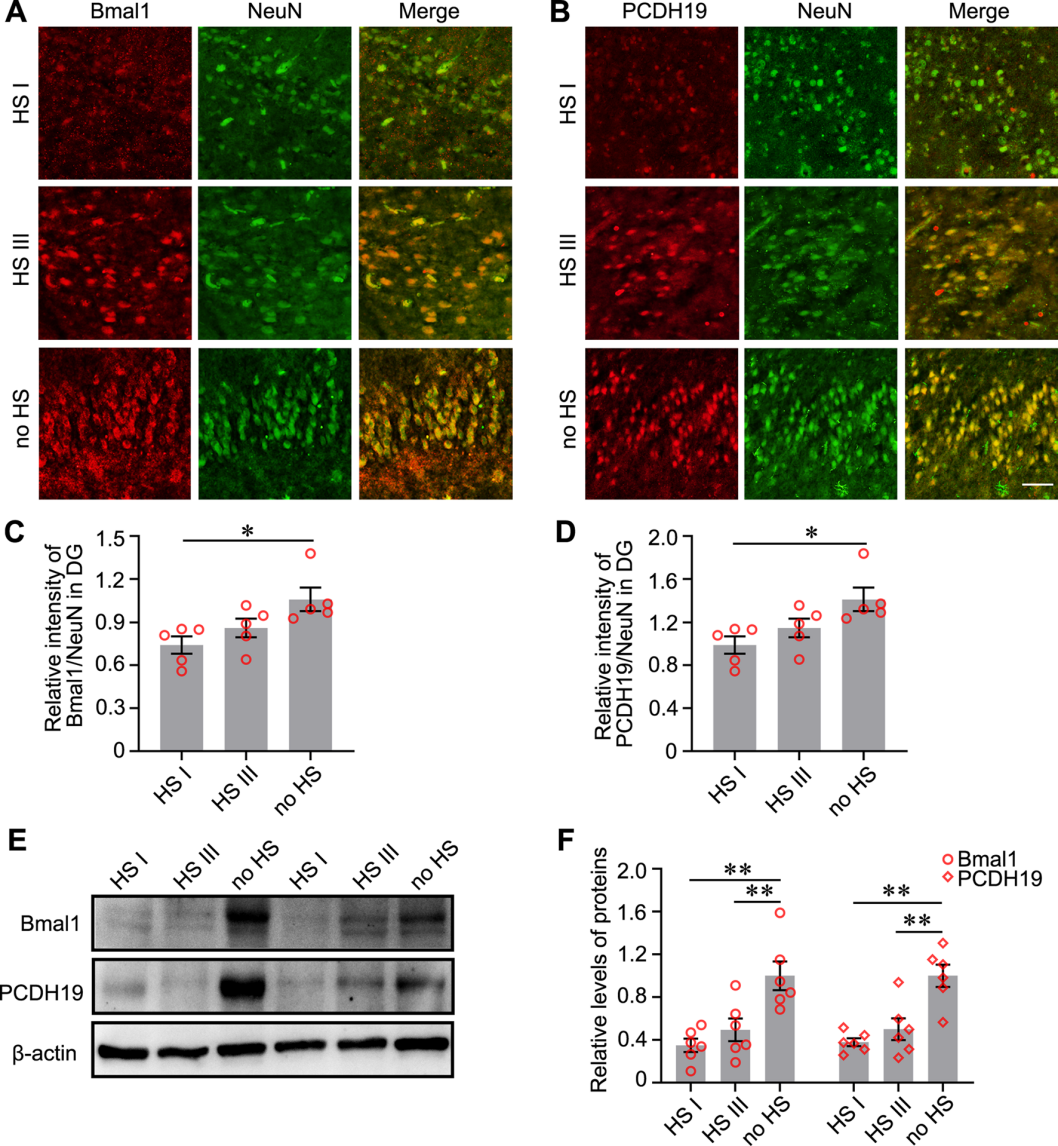
Bmal1 and PCDH19 expression in the hippocampal DG of patients with TLE. **A**, **B** Detection of the endogenous Bmal1 and PCDH19 protein (red) in DG of HS type I, HS type III, and no HS by immunofluorescent labeling. Neurons were labeled by the neuronal marker, NeuN (green). Scale bar, 100 μm. **C** Analysis of fluorescence intensity was performed using ImageJ. Differences in the relative fluorescence intensity (Bmal1 vs. NeuN) among the three groups were analyzed with one-way ANOVA, compared with no HS (HS I: 0.741 ± 0.060, HS III: 0.860 ± 0.651, no HS:1.059 ± 0.081, n = 5). **D** Differences in the relative fluorescence intensity (PCDH19 vs. NeuN) among the three groups were analyzed with one-way ANOVA, compared with no HS (HS I: 0.739 ± 0.079, HS III: 1.044 ± 0.093, no HS:1.160 ± 0.157, n = 5). **E** The levels of Bmal1 and PCDH19 protein in the hippocampus of HS type I, HS type III, and no HS by immunoblotting. **F** Differences in the levels of Bmal1 among the three groups were analyzed with one-way ANOVA, compared with no HS (For Bmal1, HS I: 0.350 ± 0.062, HS III: 0.494 ± 0.106, no HS:1.000 ± 0.135, n = 6; For PCDH19, HS I: 0.379 ± 0.037, HS III: 0.501 ± 0.102, no HS:1.000 ± 0.105, n = 6;). *p < 0.05, **p < 0.01

Hippocampal sclerosis (HS) is the common histopathological finding in patients with drug-resistant TLE [1]. The three types of hippocampal sclerosis are classified as HS International League Against Epilepsy (ILAE) type I (severe neuronal cell loss in CA1 and CA4, 50–60% granule cell loss and granule cell dispersion (GCD)), HS ILAE type II (CA1 predominant neuronal cell loss and GCD, but usually lack severe granule cell loss) and HS ILAE type III (CA4 predominant neuronal cell loss and 35% granule cell loss) [1]. In the present study, Bmal1 and PCDH19 were detected in DG of hippocampal sclerosis tissues (HS type I and III) and the tissues without hippocampal sclerosis (no HS) using immunofluorescence staining. In the DG, the intensities of Bmal1/NeuN and PCDH19/NeuN were reduced in the HS type I group were significantly reduced compared with no HS group (Fig. 6A–D). The intensity of Bmal1/NeuN and PCDH19/NeuN in the HS type III group were reduced in the HS type III group compared those with no HS group, although the difference was not statistically significant. Furthermore, the level of Bmal1 and PCDH19 in HS type I and HS type III were decreased, compared with no HS group (Fig. 6E, F).

## Discussion

In the present study, we found that Bmal1 protein was reduced in the hippocampal DG and CA1 of mice with TLE. Neuron-specific knockout of bmal1 in DG of Bmal1^flox/flox^ mice lowers the threshold of pilocarpine-induced seizures. Using high-throughput sequencing and western blotting, the downstream gene PCDH19 regulated by Bmal1 was first identified and then detected in the hippocampus of epileptic mice. Furthermore, expression of Bmal1 and PCDH19 were detected in the HS type I, HS type III, and no HS. Levels of the Bmal1 and PCDH19 proteins were decreased in the DG of HS type I and HS type III groups compared with those in the no HS group. These data suggest that Bmal1 and PCDH19 may be involved in pathogenesis of TLE.

Clock genes and CCGs not only control rhythmic physiological activities such as sleep and hormone secretion but are also involved in neurodegenerative diseases, such as Alzheimer’s disease and Parkinson’s disease [25, 26]. In previous studies, levels of the Bmal1, CLOCK, Cry and Per mRNAs were confirmed to decrease after drug-induced and electrically stimulated seizures in animal models [16]. The threshold of seizures induced by electrical stimulation in Bmal1 knockout (KO) mice was shown to be lower than that in controls [17]. In the study, the authors established mice with a systemic knockout of the Bmal1 gene, and thus they were unable to determine which organs or tissues with Bmal1 knockout directly affected seizures. In the present study, the role of Bmal1 in epileptogenesis and seizures for TLE was examined in Bmal1^flox/flox^ mice in dentate gyrus (DG). Bmal1 expression was decreased in the DG of mice and patients, and neuron-specific knockout of Bmal1 in the DG of Bmal1^flox/flox^ mice significantly shortened the latency for seizures. Thus, Bmal1 may be involved in the pathophysiology of TLE. In our study, changes in Bmal1 expression mainly occurred in the CA1 and DG neurons. As such, we did not perform knockout and functional verification of Bmal1 in hippocampal astrocytes. Alterations in the function of astrocytes caused by Bmal1 deficiency may be involved in the pathogenesis of TLE. Astrocyte-specific Bmal1 deletion induces astrocyte activation and inflammatory gene expression in vitro and in vivo and alters circadian locomotor behavior and cognition through GABA signaling in mice [14, 18]. Astrocyte activation and gliosis are one of the common pathological symptoms of TLE [27].

Currently, the molecular mechanism of Bmal1 involved in epileptogenesis has not been reported. In the present study, using high-throughput sequencing, 25 up-regulated or 19 down-regulated mRNAs in Bmal1 cKO mice were identified (FDR ≤ 0.05). As one of the candidate genes, PCDH19 is a cell adhesion molecule belonging to the cadherin family. It is expressed at high level in the CNS, especially in limbic and cortical areas [24]. PCDH19 mutations result in an epileptic syndrome known as EIEE9 (OMIM # 300088). A mechanism of cellular interference has been suggested, where the coexistence of neurons expressing wild-type (WT) or mutant PCDH19 disrupts cell–cell interactions [28]. PCDH19 downregulation has been shown to bind and regulate GABA_A_Rs kinetics and increase the frequency of action potential firing [23]. PCDH19 downregulation in rat hippocampal neurons also affects the dendrite morphology [29]. Interestingly, Clock^flox/flox^ mice with conditional deletion of the Clock gene in excitatory neurons also show specific spine defects and increased excitability [11]. Because Bmal1 and Clock are involved in transcription in the form of Bmal1:Clock complex, these indicate that abnormal expression of Bmal1 and Clock in neurons may cause similar phenotypes.

In epilepsy, DG cells form excessive de novo excitatory connections and recurrent excitatory loops, leading to the amplification and propagation of excessive recurrent excitatory signals [30]. Granule cells aggregate excitability has the potential to provide a therapeutic target [31]. In the present study, expression of Bmal1 was significantly reduced in DG of patients and mice with TLE and lowered the seizure threshold. Therefore, the DG was chosen as the target for Bmal knockout with AAV.

Clinical and animal experiments have shown that patients and animals with TLE show a 24-h non-uniform distribution of seizure occurrence [7, 32]. These suggest that the seizures of TLE may be associated with the circadian rhythms. Two hypotheses have been proposed regarding seizures associated with TLE (1) Rhythmic activity of molecules causes an increase in excitability periodically exceed the seizure threshold, displaying the behavioral seizures. For example, A-type potassium currents (IAs) exhibit a diurnal rhythm and regulat the spontaneous action potential firing in SCN during the transitions between day/night [33]. Slc6a1 (Gat1) and Slc6a11 (Gat3) control the reuptake of presynaptic GABA, while Clock Gene Rev-erbα positively regulates Slc6a1 and Slc6a11 expressions [9]. (2) Oscillation of neuronal excitability in the suprachiasmatic nucleus (SCN) modulates the rhythmic excitability in the hippocampus via neural projections [34]. Previous studies have found that Bmal1 expression level in the hippocampus still exhibit the circadian rhythmic oscillation in epilepsy [16]. Although the connection of nerve fibers between the suprachiasmatic nucleus and the dentate gyrus are not clear, the circadian rhythmic activity of DG has been reported in TLE [35, 36]. Based on these results, decreased expression of Bmal1 and PCDH19 in DG may be related to changes in the oscillation of neuronal excitability.

However, this study has some limitations. We did not evaluate the effects of Bmal1 KO in the SCN on epileptogenesis and seizures of TLE. We have not yet determined changes in the expression of circadian rhythm-related molecules in SCN in individuals with TLE. The role of Bmal1 in the SCN in individuals with TLE will continue to be explored in the future.

In conclusion, we reveal a new biological function for Bmal1 in epileptogenesis and seizures of TLE and identify a downstream gene regulated by Bmal1. Our research findings will promote the development of chronotherapy for TLE based on the chronobiology of spontaneous seizures. A more detailed understanding of the roles of Bmal1 and other Clock genes in the brain is required and may provide novel insight into the mechanism underlying epileptogenesis and seizures in patients with TLE.

## Supplementary Information

**Additional file 1: Table 1.** Clincal Features of Patients with Intractable Temporal Lobe Epilepsy.

## Data Availability

The datasets used and analyzed in this study are available from the corresponding authors on reasonable request.
