# Supplementary material for: Decreased expression of the clock gene Bmal1 is involved in the pathogenesis of temporal lobe epilepsy
Source: Mol Brain. 2021 Jul 14;14:113. doi: 10.1186/s13041-021-00824-4 (PMC8281660; doi:10.1186/s13041-021-00824-4)
Supplement: Supplementary file 1 — Additional file 1: Table 1. Clincal Features of Patients with Intractable Temporal Lobe Epilepsy. [file 13041_2021_824_MOESM1_ESM.docx]

**TABLE 1. Clincal Features of Patients with Intractable Temporal Lobe Epilepsy** *****

| Gender | Age (years) | Epilepsy duration ( years) | Seizure type | Seizure frequency | Resected tissue | Pathology | Postoperative outcome |
| --- | --- | --- | --- | --- | --- | --- | --- |
| M | 20 | 6 | SGS | 1/month | LTB;H | Gliosis | II |
| M | 43 | 20 | SGS | 2/month | LTB;H | Gliosis | I |
| M | 20 | 2 | SGS | 1/month | RTB;H | Gliosis | Ⅱ |
| F | 13 | 3 | SGS | 15/month | RTB;H | Gliosis | II |
| M | 15 | 5 | SGS | 2/month | RTB;H | Gliosis | I |
| M | 32 | 9 | CPS | 2/month | LTB;H | Gliosis | Ⅰ |
| M | 21 | 19 | SGS | 60/month | RTB;H | Gliosis | Ⅰ |
| M | 22 | 0.17 | CPS | 4/month | RTB;H | Gliosis | Ⅰ |
| F | 21 | 10 | CPS | 1/month | LTB;H | Gliosis | Ⅰ |
| F | 26 | 7 | CPS | 45/month | RTB;H | Gliosis | Ⅰ |
| F | 22 | 4 | SGS | 160/month | RTB;H | Gliosis | I |
| M | 18 | 5 | CPS | 1/month | RTB;H | Gliosis | Ⅰ |
| M | 35 | 20 | SGS | 3/month | LTB;H | Gliosis | II |
| F | 13 | 0.33 | SGS | 1-3/day | RTB;H | Gliosis | I |
| F | 26 | 11 | SGS | 1/month | RTB;H | Gliosis | I |
| M | 39 | 14 | SGS | 3-4/month | LTB;H | Gliosis | II |
| F | 30 | 7 | SGS | 2-3/month | LTB;H | Gliosis | I |
| M | 23 | 7 | SGS | 15/month | LTB;H | Gliosis | I |

***SGS**, secondarily generalized seizures; **CPS**, complex partial seizures; **LTB**, left temporal lobe; **RTB**,right temporal lobe; **H**, hippocampus; **FCD**, focal cortical dysplasia; **Postoperative outcome**, Engel’s class.
